# Analysis of Surface Extraction Methods Based on Gradient Operators for Computed Tomography in Metrology Applications

**DOI:** 10.3390/ma11081461

**Published:** 2018-08-17

**Authors:** Sinué Ontiveros, Roberto Jiménez, José A. Yagüe-Fabra, Marta Torralba

**Affiliations:** 1 Department of Industrial Engineering, Autonomous University of Baja California, 21100 Mexicali, Mexico; sinue.ontiveros@uabc.edu.mx; 2 Centro Universitario de la Defensa, A.G.M. Carretera Huesca s/n, 50090 Zaragoza, Spain; rjimenez@unizar.es (R.J.); martatg@unizar.es (M.T.); 3 I3A, Universidad de Zaragoza, María de Luna 3, 50018 Zaragoza, Spain

**Keywords:** computed tomography, surface extraction, Canny algorithm, Deriche algorithm

## Abstract

Among the multiple factors influencing the accuracy of Computed Tomography measurements, the surface extraction process is a significant contributor. The location of the surface for metrological applications is generally based on the definition of a gray value as a characteristic of similarity to define the regions of interest. A different approach is to perform the detection or location of the surface based on the discontinuity or gradient. In this paper, an adapted 3D Deriche algorithm based on gradient information is presented and compared with a previously developed adapted Canny algorithm for different surface types. Both algorithms have been applied to nine calibrated workpieces with different geometries and materials. Both the systematic error and measurement uncertainty have been determined. The results show a significant reduction of the deviations obtained with the Deriche-based algorithm in the dimensions defined by flat surfaces.

## 1. Introduction

Today’s industry increasingly demands faster and more precise systems and metrological procedures, with better economics of measurement processes and, in some cases, multifunctionality. In recent years, Computed Tomography (CT) has been acquiring an important role and is emerging as a valid option in metrological applications, being the subject of numerous investigations [1,2,3,4,5]. However, a considerable amount of work is still needed to ensure traceability in the measurements. For today’s industry, CT is the only technology available to carry out a wide variety of analyses simultaneously and non-destructively [4]. While it is true that CT has great advantages, it is also true that it has some significant limitations.

Among the multiple factors of influence in CT, surface extraction is a significant contributor. This process is mainly affected by the methodology used to locate the surface and by the algorithm used in the process of forming it. The location of the surface for metrological applications is generally based on the definition of a gray value as a characteristic of similarity to define the regions of interest [6]. This gray value threshold is mainly dependent on the properties of the material, the thickness of the piece, and the radiation intensity that is used in the CT data acquisition; therefore, a standard value cannot be determined as it is done in medical applications. A typical method for identifying the threshold value is the ISO 50% method, which has been shown on numerous occasions to result in a displaced surface [7]. Another method uses the histogram of individual cross-sectional slices with a separate determination of the threshold value for each stack, although its use is only recommended in CT images of parts made of a single material [8]. The surface extraction procedure presented in [9] is a method that requires the calibration of a reference workpiece to determine a certain correction for air-material threshold values on the gray scale of the CT volume. An alternative method is based on the local or dynamic threshold, in which a threshold value can be defined for each of the elements or zones of the tomographic volume [10]. There are other investigations focused on the determination of the surface in multi-material pieces [11,12], but they are also based on the determination of a gray value threshold as a starting point and depend on the user’s experience for its correct use.

A second source of influence comes from the algorithm used in the formation of the surface, a process for refining the surface location to obtain sub-voxel determination. There are several algorithms that can be employed to achieve this refinement and they can be grouped into three categories: reconstruction algorithms, in which the surface is reconstructed from the discrete values obtained in the surface location step [13]; interpolation algorithms, where the gradient function is interpolated with a neighborhood of the local maximum [14]; and algorithms based on the calculation of moments, which use information of the numerical properties of the image to calculate the final position of the surface (generally, this information comes from the derivative of the gradient of the image [15]).

An approach different to the use of threshold values is to perform the detection or location of the surface based on the discontinuity or gradient. According to this approach, an edge or surface of an image is a significant local change in the intensity of the image that is usually associated with a gray level discontinuity. This principle allows the derivation of a single operator shape that is optimal at any scale. The optimal detector has a simple approximate implementation in which edges are marked at the maximum of a Gaussian-smoothed gradient function [16]. This type of technique uses local operators based on different discrete approximations of the first and second derivatives of the gray levels of the image. In metrological applications, it has been demonstrated that an algorithm whose development is based on the Canny algorithm provides reduced measurement deviations than an algorithm based on a dynamic threshold and is capable of offering similar or reduced measurement uncertainty [17].

In this investigation, a comparison is made between two discontinuity- or gradient-based surface extraction procedures: a Canny-based algorithm and a Deriche-based algorithm. Despite the observed benefits of the algorithm based on Canny, it requires a high computational cost in the first preliminary edge detection step. In addition, saving the complete results of step one requires a significant use of memory. Therefore, in order to optimize the total computational time, the subsequent “Sub-voxel refinement” is computationally lighter, by taking advantage of the information provided by the preliminary edge detection step. The Deriche algorithm allows a faster preliminary edge detection, which permits the “Sub-voxel refinement” step to be more exhaustive, and consequently, may improve the precision obtained. The preliminary edge detection looks for specific gradient values for each surface point, which are calculated along the surface normals of the workpiece. Performing the “Sub-voxel refinement” along the surface normals can increase the independence of the results with respect to the orientation of the workpiece during scanning.

This study compares the performance of the new approach based on Deriche with the performance of the Canny algorithm for different surface types (e.g., spheres, cylinders, planes, and free forms). To perform this analysis, both algorithms have been applied to nine workpieces with different geometries (including complex geometries) and materials, calibrated standard parts, commercial workpieces, and multimaterial workpieces. A complete measurement must have a statement of uncertainty. Therefore, uncertainty from both methods is determined by comparison to calibrated workpieces. A cone beam CT system has been used for measurements in this study.

The article is organized as follows: First, the algorithms used and the results of the measurements (deviation and measurement uncertainty), together with the characteristics of the workpieces and the measurement systems used, are described in Section 2. The results obtained are shown in Section 3. Finally, the conclusions obtained are explained.

## 2. Materials and Methods

### 2.1. Surface Extraction Procedure

In this work, a new surface extraction algorithm based on the Deriche operator is presented. In order to evaluate its features, the new algorithm will be compared with the Canny algorithm [17].

#### 2.1.1. 3D Adapted Deriche Algorithm

The 3D adapted Deriche algorithm is a novel approach to surface extraction and is based on the work of Rachid Deriche [18]. This algorithm is based on the principles of detection quality (all existing edges should be marked and no false detection should occur), accuracy (the marked edges should be as close to the edges in the real image as possible), and unambiguity (no multiple responses to one edge in the real image should occur). The Deriche segmentation algorithm was developed for use in two-dimensional images and, despite its robust implementation, must be adapted to three-dimensions for use in CT. Furthermore, the use of CT for measurements requires sub-voxel determination.

Taking this into account, the developed algorithm implements the three following steps:

- Preliminary edge detection.
- Sub-voxel refinement.
- Suppression of no-maximum points.

**Figure 1:**
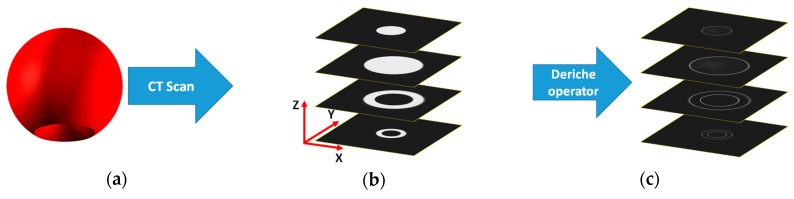
Preliminary edge detection process: (**a**) Scanned part, (**b**) image slices from 3D volume, and (**c**) Deriche edge detection according the three main directions.

These three stages are explained below:

1. Preliminary edge detection: In this first step, a gradient operator adapted from the Deriche operator is applied. In this adaptation (Figure 1), typical filtering is omitted to reduce the computational cost and avoid loss of information. Therefore, the recursive operator used is the following:

(1) $$\gamma_{ijk}^{+}=X_{ijk-1}+2\cdot e^{-\alpha }\cdot \gamma_{ijk-1}^{+}-e^{-2\cdot \alpha }\cdot \gamma_{ijk-2}^{+}$$

(2) $$\gamma_{ijk}^{-}=X_{ijk+1}+2\cdot e^{-\alpha }\cdot \gamma_{ijk+1}^{-}-e^{-2\cdot \alpha }\cdot \gamma_{ijk+2}^{-}$$

(3) $$\theta_{ijk}=-{\left(1-e^{-\alpha }\right)}^{2}\cdot (\gamma_{ijk}^{+}+\gamma_{ijk}^{-})$$

where *X_ijk_* is the gray value for the voxel *ijk* and *α* is a parameter of the gradient operator, the value of which was determined experimentally to be equal to 3.

Applying this operator in each of the three orthogonal *XYZ* directions of the 3D volume (Figure 1b), the value of the gradient of each voxel is obtained according to those same directions ($\theta_{ijk}^{X},\;\theta_{ijk}^{Y},\theta_{ijk}^{Z}$). Using Equation (4), the three values are combined to obtain a single representative value of the gradient for each point of the volume (Figure 1c).

(4) $$\theta_{ijk}^{XYZ}=\left|\theta_{ijk}^{X}\right|+\left|\theta_{ijk}^{Y}\right|+\left|\theta_{ijk}^{Z}\right|$$

2. Sub-voxel refinement: The Deriche and Canny algorithms, as proposed by their authors, define the surface points of the part only according to the coordinates of the voxels that have a maximum value. Sub-voxel determination of the surface position can be achieved using gradient information of the neighborhood voxels. In this manner, the material transition point can be localized to within 0.01 voxels.

The procedure is as follows: the elements of the matrix $\theta_{ijk}^{XYZ}$ with the largest values are taken. As described in the previous step, these values were calculated taking into account only the three main *XYZ* directions. Depending on the orientation of the surface with respect to the reference system, the limitation of preliminary edge detection for the three coordinate axis directions can result in a shift of the obtained contour points and, consequently, a reduced dimensional performance. This problem is solved by applying this sub-voxel refinement along the surface normal.

**Figure 2:**
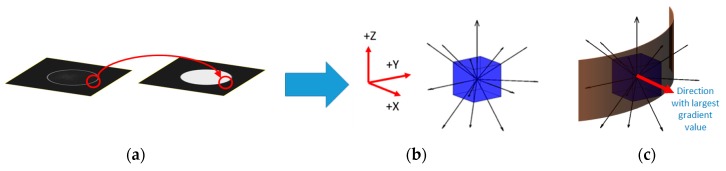
Sub-voxel refinement process: (**a**) Local maximum position detected, (**b**) calculation of the gradient along 13 directions, and (**c**) normal surface obtained from the largest gradient value direction to calculate the center of gravity.

To calculate the gradient through the surface normal or close to it, the developed Deriche algorithm is applied along 13 directions: The three main directions (+*X*, +*Y*, +*Z*) and the ten diagonals between them are shown in Figure 2b. The value of the gradient in each of those directions will be calculated within a search window of a fixed size and using Equations (1)–(3). The direction with the largest gradient value is considered to be approximately equal to the surface normal (Figure 2c). Once the surface normal is estimated, gradient values are obtained for each voxel in the search window. The center of gravity of the search area gradients is calculated and used to define the *XYZ* surface position to less than a voxel, as shown in Equation (5).

(5) $$X=\frac{\sum_{i=1}^{i=n}\;\left(X_{i}\cdot \theta_{x}\right)}{\sum_{i=1}^{i=n}\;\left(X_{i}\right)}$$

where *θ_x_* is the gradient value of each voxel along the surface normal, and *X_i_* is the respective *XYZ* position of each voxel.

**Figure 3:**
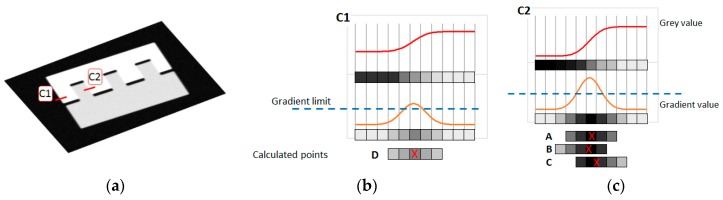
Suppression of no-maximum points in multi-material parts: (**a**) Profile; (**b**) situation where the right gradient limit value generates only one point; (**c**) situation where the same gradient limit generates several close points with high gradient values, justifying the need for non-maximum suppression.

3. Suppression of no-maximum points: In both the Canny and Deriche algorithms, the objective of this step is to identify the voxels contiguous to other voxels with higher values. While in the Canny algorithm, those voxels with lower values were eliminated, in the Deriche algorithm, this information is used to perform sub-voxel refinement. Therefore, the objective in this phase is both to keep the value of the voxels in the matrix $\theta_{ijk}^{XYZ}$ (i.e., those with higher values) and to not generate new points too close to other voxels with higher gradient values, which is a risk when those contiguous voxels with lower values are used. Therefore, once a point has been generated based on the previous step, those points which, following the direction of the maximum local slope, are at a distance less than a predetermined limit, will be marked. These marked voxels will be ignored when generating new points regardless of their value (Figure 3). This step is especially important in multi-material parts, where the gradient values may exhibit significant variations depending on the interface surface (e.g., air-part or part-part) (Figure 3a). Since only one gradient limit is used in the previous step, the right value for the part-part interface surfaces (Figure 3b) would generate, in this step, several very close points in the air-part interface surfaces (see Figure 3c). Therefore, it must be considered that, when the wall thickness or gaps between elements of very few voxel sizes are expected, this limit should be set carefully, due to the risk of eliminating one of the two surfaces.

#### 2.1.2. Canny Algorithm (CA)

The Canny-adapted algorithm for the 3D surface extraction process was developed by the authors and is fully explained in [17]. The implementation of this algorithm follows the same three steps as in the Deriche algorithm, but with two main differences:

1. The preliminary edge detection in the Canny algorithm is based on the application of the Canny filter to the images along each of the three Cartesian directions, using a 1 × 10 convolution mask, oriented along the direction. This process leads to a precise determination of the gradient, but it requires significant computational resources.
2. The second main difference is in the sub-voxel refinement step. In the Deriche algorithm, the point is calculated along a direction perpendicular to the part surface, while in the Canny algorithm, the calculation of the optimal position of the point with sub-voxel definition is carried out by applying Equation (6):
  (6) $$X^{\prime }=\frac{\sum_{i=1}^{i=3}\left(X_{i}•G_{X,i}\right)}{\sum_{i=1}^{i=3}G_{X,i}};Y^{\prime }=\frac{\sum_{j=1}^{j=3}\left(Y_{j}•G_{Y,j}\right)}{\sum_{j=1}^{j=3}G_{Y,j}};Z^{\prime }=\frac{\sum_{k=1}^{k=3}\left(Z_{k}•G_{Z,k}\right)}{\sum_{k=1}^{k=3}G_{Z,k}}$$
  where *X_i_*, *Y_j_*, and *Z_k_* are the coordinates of the voxels inside the search window; and *i*, *j*, and *k* are the voxel indices within the search window. *G*_*X*,*i*_, *G*_*Y*,*j*_, and *G*_*Z*,*k*_, with possible values from 0 to 65,535, i.e., 16 bits, are the gray value transitions obtained in the preliminary surface detection phase for the *X*, *Y*, and *Z* directions, respectively. This refinement is carried out separately and independently along the three directions, providing the three dimensional coordinates of each surface point. In order to obtain each of the coordinates, only the 3D image along that direction, obtained from the previous step, is used.

### 2.2. Workpieces

**Figure 4:**
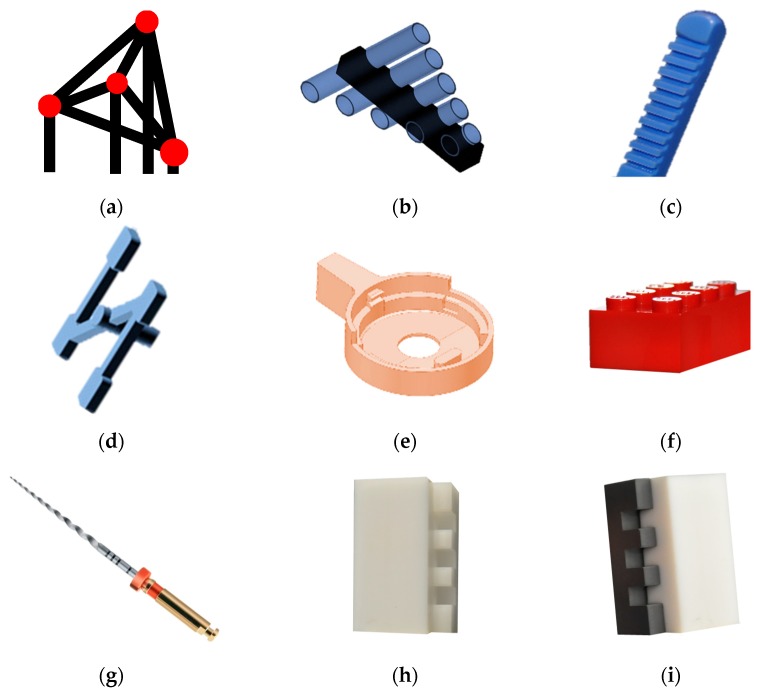
Work pieces: (**a**) CT Tetrahedron, (**b**) Pan Flute, (**c**) Step Gauge, (**d**) Dog bone, (**e**) Toggle, (**f**) Lego, (**g**) Endodontic file, (**h**) Multi-material TEF-POM, and (**i**) Multi-material TEF-PVC.

In this study, a total of nine calibrated workpieces with different characteristics and materials have been used (Figure 4). Pieces with primitive geometries (spheres), pieces with dimensions dependent on each other (outer and inner cylinder), with parallel planes (grooves), with different materials, and different sizes have been chosen. In addition, a piece with a complex geometry and multimaterial pieces have been used to assess the behavior of surface extraction procedures in these cases. With the pieces used during the investigation, a wide spectrum of geometric and material characteristics are covered, which makes it possible to ensure the generality of the results obtained.

**Figure 5:**
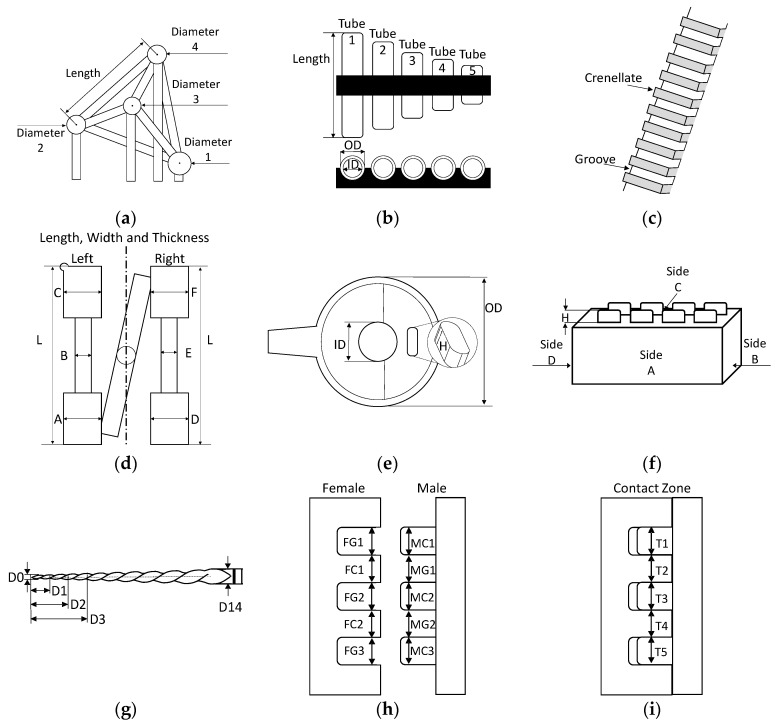
Work piece dimensions: (**a**) CT Tetrahedron, (**b**) Pan Flute, (**c**) Step Gauge, (**d**) Dog bone, (**e**) Toggle, (**f**) Lego, (**g**) Endodontic file, (**h**) male and female in TEF-POM and TEF-PVC, and (**i**) contact zone in TEF-POM and TEF-PVC.

CT Tetrahedron: This part is called the “CT Tetrahedron” (Figure 4a) and is part of the group of parts used in the “CT Audit” intercomparison [19]. The CT tetrahedron consists of four synthetic ruby spheres held in place by a structure of carbon fiber: the centers of the four spheres coincide with the vertices of a tetrahedron. The dimensions analyzed in this piece are the diameters of each sphere (D) and the distance between them (L). The nominal dimensions are: D1 = 5 mm, D2 = 4 mm, D3 = 4 mm, D5 = 3 mm, and L = 25 mm (Figure 5a). This piece has been manufactured and calibrated by the University of Padova [19].

Pan Flute: The Pan Flute Gauge (Figure 4b), like the CT Tetrahedron, was also used in the intercomparison “CT Audit”. This piece consists of five glass tubes supported by a carbon fiber frame. The dimensions analyzed are the inside diameters (ID), outside diameters (OD), and the length (L) of each of the tubes. The five tubes have the same nominal diameters, ID = 1.5 mm and OD = 1.9 mm, while the tube lengths are L1 = 12.5 mm, L2 = 10.0 mm, L3 = 7.5 mm, L4 = 5.0 mm, and L5 = 2.5 mm (Figure 5b). The pan flute has been manufactured and calibrated by the University of Padova [19].

Step Gauge: The Step Gauge (Figure 4c) is a 42 mm long slotted pattern that was specifically designed for the verification of optical systems for micro-fabrication by the Technical University of Denmark—DTU [20]. The step gauge is made of bisacryl, a material used in dental applications. In this piece, the following dimensions have been verified: The distance between each of the slots (R), the distance between each casing (A), and the passage of the slot (P) between each of the slots (Figure 5c). Each groove and casing has a nominal distance of 2 mm and the pitch between similar features is 4 mm. This part was calibrated at the University of Zaragoza.

Dog bone: This part (Figure 4d) has been manufactured by micro-injection and is used to perform an analysis of the mechanical behavior of the material. It is made of polyoxymethylene (POM), also called acetal, polyacetal, and polyformaldehyde. It is a technical thermoplastic used in precision components that require a high rigidity, low friction, and excellent dimensional stability. POM is characterized by its high strength, hardness, and rigidity [21]. Two types of dimensions have been verified for this piece: the length of the piece in four different zones on both sides of the piece (L, a, b, and c) and the thickness in six zones of the two lateral bodies (A, B, C, D, E, and F). The nominal dimensions of each of the dimensions are the following: L = 11.80 mm, a and c = 3.00 mm, b = 1.50 mm, d = 1.35 mm, and the thickness from A to F = 1.00 mm (Figure 5d).

Toggle: This part (Figure 4e) has been manufactured in liquid crystal polymer (LCP) by a micro-injection process. Three dimensions of this part have been verified: the outside diameter of the main body (D), the diameter of the central hole (d), and the height of the pillar (A) located inside (Figure 5e). The nominal dimensions are the following: D = 5.40 mm, d = 1.55 mm, and H = 0.38 mm [22].

Lego: This piece is a common block of Lego^®^ (Figure 4f) and has been used in the intercomparison of coordinate measuring machines “Video Audit” led by the University of Padova. The dimensions verified in this work piece are the following: The diameter (D) of each of the buttons, the height (A) of button 1, and the length (L) of each of the sides (LA, LB, LC, and LD). The nominal values of each dimension are the following: D = 5.0 mm, A = 1.7 mm, LA and LB = 31.0 mm, and LB and LD = 16.0 mm (Figure 5f) [23].

Endodontic file: In addition to primitive geometries, we decided to analyze the behavior of the algorithms in a piece with a complex geometry, which is why an endodontic file has been chosen (Figure 4g). It belongs to the ProTaper mechanical instrumentation system developed by the Dentsply Maillefer company. The instruments of the ProTaper system are manufactured with an Ni-Ti alloy [24]. All the pieces of this system have geometrical characteristics of great interest from the point of view of their dimensional characterization: they have a variable conicity that allows the instrument to reduce stress due to torsion, file fatigue and the possibility of fracture, the angle, and a step of the variable helix, with propellers of increasing separation as one advances in the direction of the shank of the instrument. In addition, the ProTapet endodontic files are characterized as having a triangular transversal section that is convex or rounded. The chosen piece is a file for the endodontics of model F2. This piece has a red identification ring that indicates that it is a model characterized by having a greater conicity in the tip, which decreases in the direction of the stem. The dimensions verified in the work piece are the following: (1) Length of the active part (La), which comprises the active cutting surface; and (2) diameter (D), which varies along the length of the entire file and is measured from the center of the tip to the shank of the file, with D0 being the diameter of the tip of the file, and every millimeter moving away from the file being D1, D2, D3, etc. (Figure 5g).

TEF-POM: This is a multi-material part composed of two slotted elements (male and female, Figure 4h). It is made of Polytetrafluoroethylene (PTFE) and POM. The Polytetrafluoroethylene or Teflon is considered to be one of the most versatile among plastic materials due to its many applications, for which other materials cannot be utilized. PTFE is a high temperature resistant material with a low friction coefficient and high resistance to the action of chemical agents and solvents, among other characteristics. PTFE is often considered to be a thermostable polymer and can be used in a continued process at any temperature between −200 °C and +260 °C [25]. POM is a technical thermoplastic used in precision components that require a high rigidity and excellent dimensional stability. POM is characterized by high resistance, hardness, and rigidity up to −40 °C. The most common applications are sectors such as automotive, consumer electronics, heavy loads transport applications, and in low temperatures [26].

TEF-PVC: The second multi-material piece is made of PTFE and Polyvinyl chloride (PVC) (Figure 4i). PVC is a very durable and long lasting thermoplastic construction material that can be used in a variety of applications, can be either rigid or flexible, and can be produced in a wide variety of colors. Due to these characteristics, PVC is used in many industries and is found in many popular and necessary products [27].

Both elements of parts TEF-POM and TEF-PVC have been assembled in a way in which a portion of their interface is not in direct contact. In addition, the assembly tolerance in the contact zone is an adjustment without interference, which does not deform any of the elements. The dimensions that have been evaluated are: The width of the groove (G) and the crellenate (C) and the contact zone between the two pieces (T). G, C, and T have a nominal distance of 5 mm. Therefore, 15 dimensions were assessed in total: Five in the female piece (FG1, FC1, FG2, FC2, and FG3), five in the male piece (MC1, MG1, MC2, MG2, and MC3), and five in the contact zone (T1, T2, T3, T4, and T5) (Figure 5h,i).

### 2.3. Computed Tomography System

The workpieces were measured on a cone-beam micro-CT eXplore Locus SP machine by General Electric (Boston, MA, USA). The CT machine has an X-ray source that has a power range from 50 to 90 kV, a resolution (minimum voxel size) of 8 μm, and a cylindrical work volume of 44 mm in diameter by 56 mm in height. During the scanning of the workpiece, the temperature was continuously recorded inside the machine, obtaining a temperature range of 20 ± 2 °C for all pieces. Each workpiece was measured 10 times. The selected parameters used for the CT measurements are presented in Table 1.

### 2.4. Calibration Systems

#### 2.4.1. Coordinate Measuring Machines (CMM)

**Figure 6:**
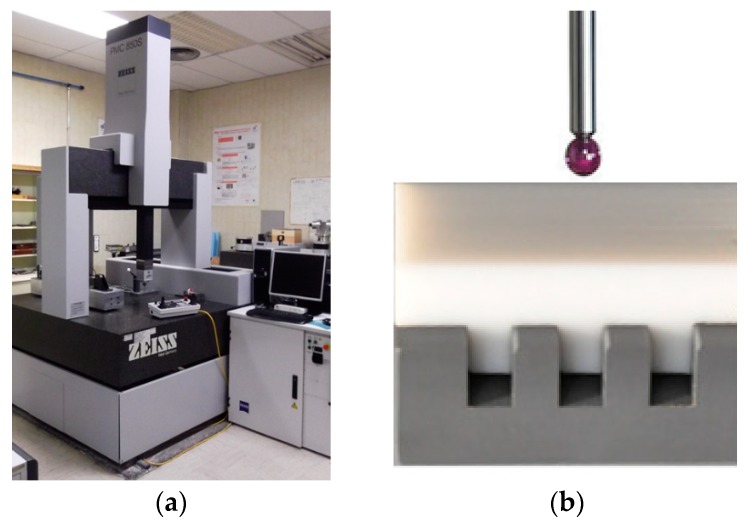
(**a**) CMM Carl Zeiss PMC 850S-CNC used for the calibration of the Step Gauge, the Lego, and the TEF-POM and TEF-PVC workpieces [28]; (**b**) image of the measuring process of the TEF-PVC workpiece with the CMM.

Two CMMs were used to calibrate the workpieces used in this study. The Step Gauge, the Lego, and the TEF-POM and TEF-PVC workpieces [28] were calibrated on a PMC 850S-CNC CMM (Figure 6) by Carl Zeiss (Oberkochen, Germany), which has a measurement volume of 850 × 700 × 600mm and a longitudinal MPE = 2.30 μm + (L/300) μm (L in mm). The probing system used was a Zeiss Vast-XT with an active scanner, allowing working with extensions of up to 500 g in weight and 500 mm in length.

The CT Tetrahedron and the Pan Flute were calibrated at the University of Padova using a tactile CMM (MPE = 1.4 + L/300 μm, with L in mm). The measurements were performed following the procedures indicated during the CT Audit inter-comparison [19].

#### 2.4.2. Optical Coordinate Measuring Machines (OCMM)

**Figure 7:**
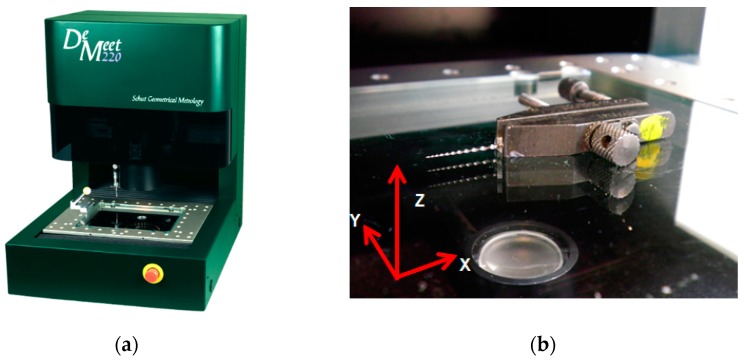
(**a**) OCMM De Meet 220 [29] used for the calibration of the Dog bone, Toggle, and Endodontic file workpieces; (**b**) image of the measuring process of the Dental file workpiece with the OCMM.

An optical coordinate measuring machine DeMeet 220 [29] has been used as a reference calibration system for the Dog bone, Toggle, and Endodontic file parts. The DeMeet 220 machine by Schut (Groningen, The Netherlands) has a measuring range of 220 mm × 150 mm × 100 mm, backlight and coaxial light, diascopic illumination with a light ring, and a magnification lens 2×. The DeMeet 220 has tele-centric optics to avoid distortion around the center of the field of view, can achieve magnifications in the range of 40× to 400×, and has a field of view of 3111 µm × 2327 µm (Figure 7).

The OCMM uncertainty for length measurements in the 100–1000 μm range was evaluated, resulting in a maximum permissible error MPE_OCMM_ = 1.7 μm (i.e., suitable for the diameter measurements of the endodontic file). For the measurements of the endodontic file with a length L > 1 mm, the maximum permissible error of the OCMM obtained is: MPE_OCMM_ = 5 μm + (L/150) μm (L in mm).

## 3. Results

### 3.1. CT Comparison Results

**Figure 8:**
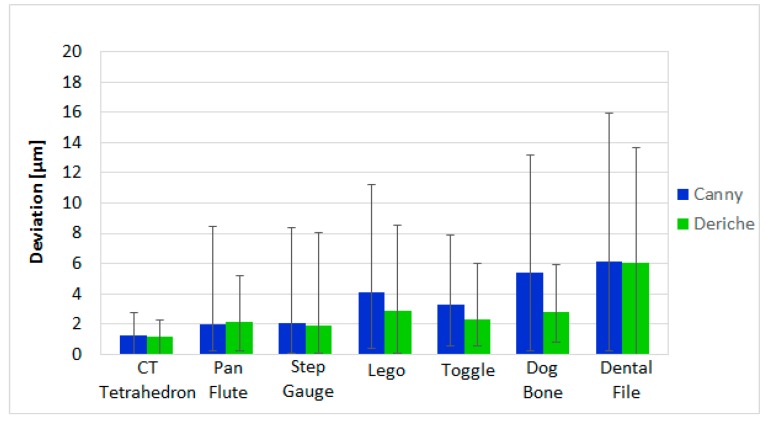
Average deviation for the Canny-based method (blue bars) and Deriche-based method (green bars) and maximum and minimum deviation (error bars) of all the part dimensions from their calibrated absolute values of single material parts.

CT measurements using each surface extraction procedure are compared to the respective reference values. In Figure 8, the average absolute deviation over 10 repeat measurements of the first seven workpieces (single material parts) is shown. This average deviation takes into account all the dimensions measured for each part. In addition, the maximum and minimum deviations of all those dimensions from their absolute calibrated values are represented by the error bars for each part. Deviations from Deriche-based surface extraction are lower in six of seven pieces when compared to the deviations from Canny-based surface extraction. The largest reduction in deviations is observed for measurements of the Toggle and Dog Bone, where the deviations are reduced by more than 50%. Deviation was 7% higher for Deriche-based surface extraction in the measurement of the Pan Flute. Pan Flute was the only workpiece where the deviation was higher for Deriche than for Canny. Absolute maximum deviations were lower with Deriche-based surface extraction for all the measured parts, observing the largest difference, equal to 7.9 μm, between the two surface extraction methods in the Dog Bone workpiece.

**Figure 9:**
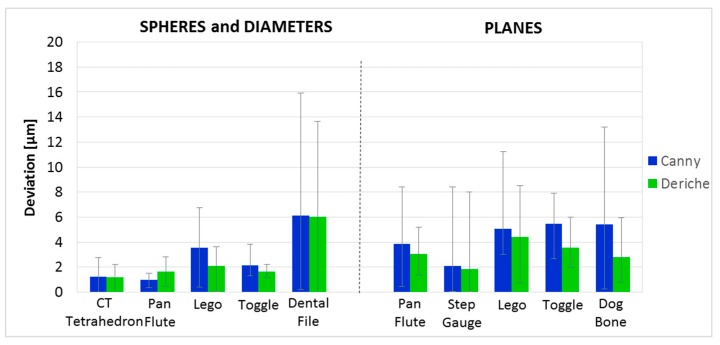
Average deviation for the Canny-based method (blue bars) and Deriche-based method (green bars) and maximum and minimum deviation (error bars) of all the part dimensions from their calibrated absolute values of single material parts grouped by dimension type.

Figure 9 shows a differentiated analysis by the type of surface. On the left side of the graph, the average deviation of spheres, diameters, or dimensions dependent on these two forms (e.g., distance between spheres) is presented, while on the right side are the dimensions dependent on planes. For dimensions related to spheres and diameters, Deriche-based surface extraction provides lower deviations in four of five workpieces. The most significant reduction is observed in the measurement of the Lego piece. With the Canny-based surface extraction algorithm, the average deviation is 3.6 μm and the maximum deviation is 7.6 μm. Meanwhile, with Deriche-based surface extraction, the average deviation is 2.1 μm and the maximum deviation is 3.6 μm. In the case of the planes-dependent dimensions, deviations are lower with Deriche-based surface extraction. The most significant case is that of the Dog Bone piece, where the average deviation with Canny-based surface extraction is 5.4 μm, while it is only 2.8 μm with Deriche-based surface extraction. Absolute maximum deviations were lower with Deriche-based surface extraction for all the measured parts and all the dimension types.

**Figure 10:**
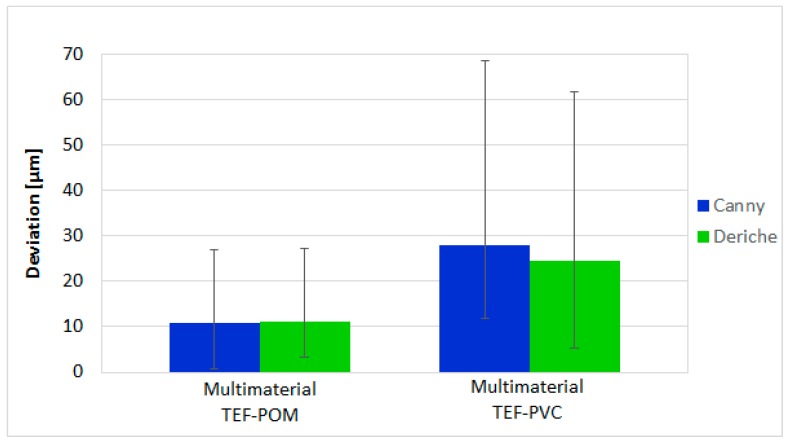
Average deviation for the Canny-based method (blue bars) and Deriche-based method (green bars) and maximum and minimum deviation (error bars) of all the part dimensions from their calibrated absolute values of multimaterial workpieces.

Figure 10 shows the results obtained in the multimaterial workpieces. In the measurement of the TEF-POM, the average deviation with the Canny-based surface extraction is only slightly lower (difference equal to 0.2 μm) than with Deriche-based surface extraction. For the TEF-POM, the maximum deviation with the Canny-based surface extraction is also slightly lower (difference equal to 0.3 μm) than with Deriche-based surface extraction. In the measurement of the TEF-PVC part, the average deviation and the maximum deviation with Deriche-based surface extraction are smaller than the same deviations with Canny-based surface extraction by 3.5 μm and 7.0 μm, respectively.

### 3.2. Uncertainty Estimation for CT Measurements

In order to evaluate the influence of the data processing on uncertainty, the most accepted standard currently available for CT sensors is applied to compare both surface extraction methods, i.e., the Verein Deutscher Ingenieure/Verband Deutscher Elektrotechniker (VDI/VDE) guideline 2630-2.1 [30]. This standard is based on the assessment of the measurement uncertainty by means of a calibrated workpiece. The expression of the uncertainty is given by Equation (7).

(7) $$U95,\mathrm{method}\left(i\right),\mathrm{piece}\left(j\right),\mathrm{measurand}\left(k\right)=2\cdot \sqrt{u_{\mathrm{cal}}^{2}+u_{p}^{2}+u_{w}^{2}+u_{b}^{2}},$$

For expanded uncertainty with a confidence interval of 95.45%, which is expressed as *U*_95_ in this study, the coverage factor is equal to 2, as shown in Equation (7). The measurement uncertainty value depends on the surface extraction method (i = Canny or Deriche); the analyzed workpiece (j = 1, ..., 7); and the measurands (k), which in this work, are mainly separated as spheres and diameters or planes. The considered error sources in the uncertainty budget are those included in the VDI/VDE 2630-2.1 standard and they are the following:

▪ The term *u*_cal_ represents the standard uncertainty of calibration of the workpiece determined by a CMS (coordinate measuring system).
▪ The term *u*_p_ is the standard uncertainty of the measurement procedure (repeatability).
▪ The term *u*_w_ is the standard uncertainty of the material and manufacturing variations of the measured process. It is specifically associated with two uncertainty sources: variations in the mechanical properties of the workpiece (*u*_w1_); and variations in the CTEs (coefficient of thermal expansion) of the workpiece (*u*_w2_). In this work, the first factor has been previously included in u_p_ (effects of material composition and shape). A rectangular statistical distribution for 20% of CTE variation has been established for the second term.
▪ The term *u*_b_ is the standard uncertainty associated with the systematic error of the measurement process: the influence of the temperature variation during the CT measurement (*u*_b1_ when ∆T = ±2 °C); and the estimation of the systematic error related to the surface detection technique: Canny and Deriche (*u*_b2_).

**Figure 11:**
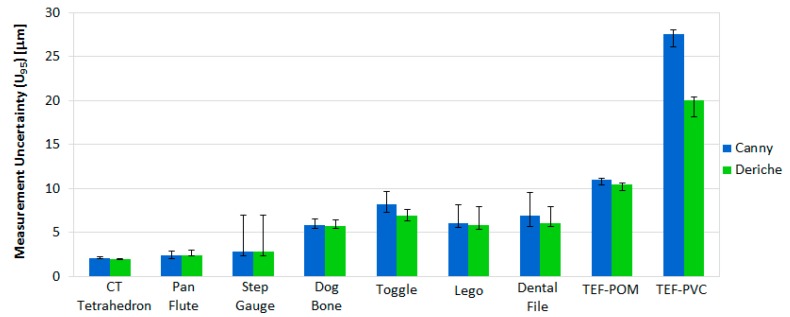
Expanded average measurement uncertainty (*U*_95_) for the Canny-based method (blue bars) and Deriche-based method (green bars), and maximum and minimum measurement uncertainties (error bars) of all the part dimensions for each item.

**Figure 12:**
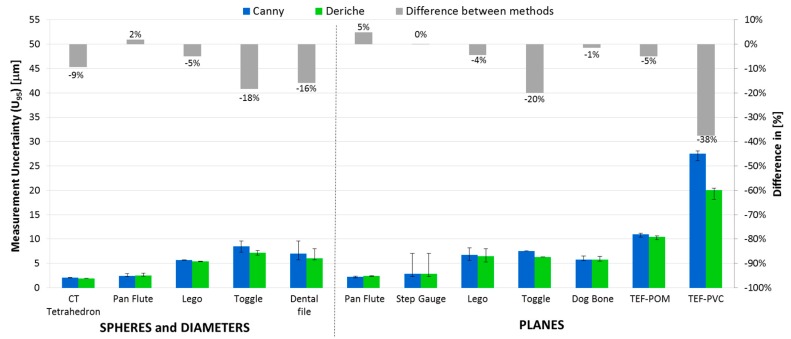
Expanded average measurement uncertainty (*U*_95_) for the Canny-based method (blue bars) and Deriche-based method (green bars), differences between them (grey bars), and maximum and minimum measurement uncertainties (error bars) of all the part dimensions for each item by dimension type.

The average expanded uncertainty, taking into account all the part dimensions, is shown in Figure 11 by workpiece and in Figure 12 by type of measured geometry in each item. The error bars in both Figures represent the maximum and minimum expanded uncertainty of all the part dimensions for each part. As can be seen in both Figure 11 and Figure 12, Deriche-based surface extraction generally provides lower average, maximum, and minimum uncertainties *U*_95_ for each item.

**Figure 13:**
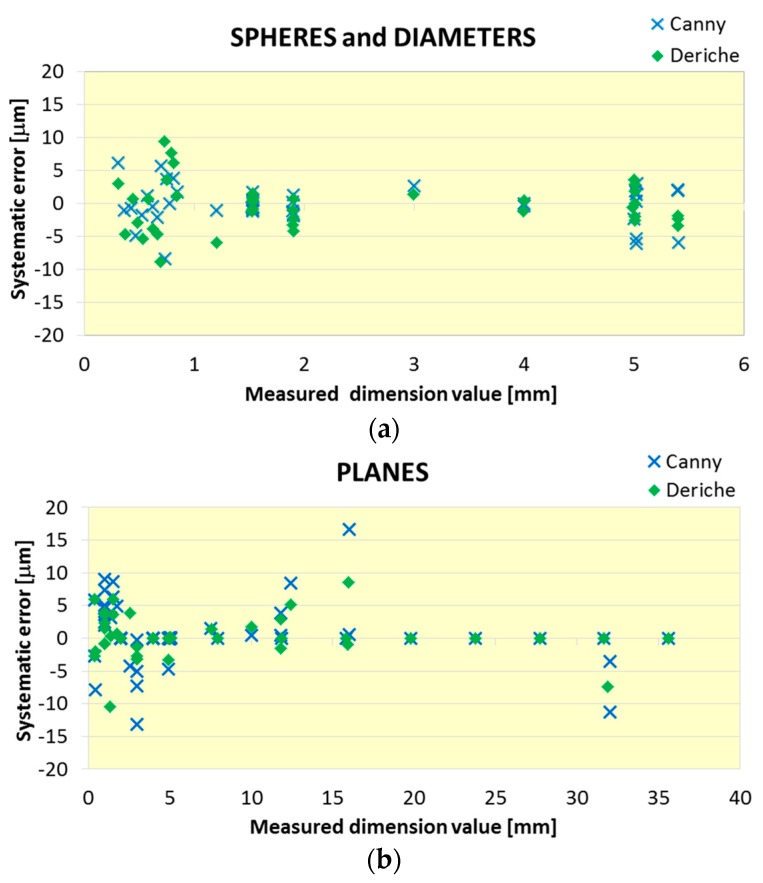
Systematic error source contribution analysis (*u*_b2_) by method and by dimension type for (**a**) spheres and diameters and (**b**) planes.

Most of the different uncertainty contributors considered in Equation (7) suppose a similar contribution in the expanded uncertainty for both Canny- and Deriche-based surface extraction. Nevertheless, the systematic error contribution (*u*_b2_) associated with the surface extraction method shows the largest difference between *U*_95_ obtained with Canny- and Deriche-based surface extraction. Additionally, *u*_b2_ constitutes 30% of *U*_95_, so a specific analysis was carried out and shown in Figure 13, where the contribution u_b2_ is represented for both methods for the two surface typologies.

Analyzing the contribution u_b2_ of both methods for the two types of surface, curves and planes, a similar behavior can be observed between both algorithms for the case of spheres and cylinders. However, for the defined dimensions between flat surfaces, the Deriche-based algorithm slightly improves the results obtained for most of the pieces. 

## 4. Discussion

The average deviations with respect to the calibrated values do not present, in a general way, large differences for the two algorithms analyzed, with only a slight improvement in the Deriche-based surface extraction algorithm. However, a clear difference between curved and flat surfaces is concluded from this analysis: the reduction of the deviations obtained with the Deriche-based algorithm is evident, with reductions as large as 50% with the Deriche-based surface extraction for flat surfaces compared to the Canny-based algorithm. Similar results were observed in measurement uncertainty. The results of uncertainty obtained for the Deriche-based algorithm are further reduced in the dimensions defined by flat surfaces.

Both results agree with the main novelty of the Deriche algorithm: the realization of the “Sub-voxel refinement” in a direction perpendicular to the surface. In flat geometries, a non-optimal orientation is more detrimental to deviations, and especially to the uncertainty obtained, in the Canny-based algorithm. In contrast, the Deriche-based algorithm reduces the effect of the orientation in which the piece was measured. 

Also noteworthy are the results of the two multi-material pieces, both with defined dimensions between flat geometries. In this case, although in the TEF-PVC workpiece the uncertainties are larger than in any other, so is the reduction that is achieved by applying the algorithm based on Deriche. This multi-material piece is made of two materials with similar attenuation coefficients, Teflon and PVC, which makes it more difficult to distinguish them in the contact area. This fact would justify the greater difficulty in its precise measurement, but it must also be highlighted that the best behavior of the Deriche-based algorithm is displayed in these difficult conditions. 

## 5. Conclusions

In this paper, an adapted 3D Deriche algorithm based on gradient information has been presented and compared with a previously developed adapted Canny algorithm for different surface types. Both algorithms have been applied to the measurement of nine calibrated workpieces with different geometries and materials. Both the systematic error and measurement uncertainty have been determined; a significant reduction of the deviations obtained with the Deriche-based algorithm in the dimensions defined by flat surfaces and a slight improvement in the dimensions defined by spheres and diameters is observed. Therefore, the use of this algorithm could prove pivotal in reducing systematic errors and uncertainty in CT measurements. 

The approach used in the Deriche-based algorithm can, in certain cases, be a more efficient alternative for surface extraction. In the Deriche-based surface extraction, the calculations made in the “Sub-voxel refinement” step are independent of the previous step, which does not happen in the Canny algorithm. This may improve the efficiency of the measuring process implemented with a Deriche-based algorithm. For example, in order to use the Deriche-based surface extraction, the information of the “Preliminary edge detection” could be directly obtained from nominal information of the piece supplied (e.g., in CAD format). Provided it is possible to perform an alignment of that CAD model with the position of the piece in the scan, the use of nominal information could further improve the efficiency of Deriche-based surface extraction. Another possibility would be to perform the “Preliminary edge detection” using other types of algorithms more efficient but with limitations in terms of accuracy. This would be the case for the techniques of threshold ISO 50%; although it is demonstrated that they quickly obtain a fast extraction of surfaces, it is also demonstrated that the obtained precision is inferior to the one of the gradient techniques. After the “Preliminary edge detection” step with the threshold ISO 50%, the Deriche-based algorithm “Sub-voxel refinement” and “Suppression of no-maximum points” could be applied to improve the performance.

## Figures and Tables

**Table 1:** CT scanning parameters.

Parameter	CT Tetrahedron	Pan Flute	Step Gauge	Dog Bone	Toggle	Lego	Endodontic File	TEF-POM	TEF-PVC
Voltage (kV)	90	90	45	80	80	45	90	45	45
Current (µA)	80	80	120	95	95	120	80	120	120
Angular indexing step (degrees)	0.2	0.35	0.4	0.4	0.4	0.4	0.4	0.3	0.3
Voxel size (µm)	45.5	16.5	47	8	8	34	28	28	56

## Nomenclature

CMM	Coordinate measuring machine
CMS	Coordinate measuring system
CT	Computed Tomography
DTU	Danmarks Tekniske Universitet (Technical University of Denmark)
ID	Inside Diameters
LCP	Liquid Crystal Polymer
OCMM	Optical Coordinate Measuring Machine
OD	Outside Diameters
POM	Polyoxymethylene
PTFE	Polytetrafluoroethylene (Teflon^®^)
PVC	Polyvinyl chloride
TEF-POM	Multi-material part made of PTFE and POM
TEF-PVC	Multi-material part made of PTFE and PVC
VDI/VDE	Verein Deutscher Ingenieure/Verband Deutscher Elektrotechniker
